# Diagnosis of low bone mass in CKD-5D patients 

**DOI:** 10.5414/CN108708

**Published:** 2015-11-20

**Authors:** Gustav A. Blomquist, Daniel L. Davenport, Hanna W. Mawad, Marie-Claude Monier-Faugere, Hartmut H. Malluche

**Affiliations:** 1 Department of Radiology,; 2 Department of Surgery, and; 3 Division of Nephrology, Bone and Mineral Metabolism, University of Kentucky, Lexington, KY, USA

**Keywords:** chronic kidney disease, dialysis, renal osteodystrophy

## Abstract

Background and objectives: Currently, there is no consensus whether dual-energy X-ray absorptiometry (DXA) or quantitative computed tomography (QCT) can be used to screen for osteoporosis or osteopenia in CKD-5D patients. This study uses iliac bone histology, the “gold standard” for bone volume evaluation, to determine the utility of DXA and QCT for low bone mass screening in CKD-5D patients. Patients and methods: A cross-sectional study of patients with CKD-5D employing iliac crest bone biopsies to assess bone volume by histology and comparing results to bone mineral density (BMD) measurements of the hip and spine by DXA and QCT. Pearson’s correlation, linear regression, and receiver operating characteristics curve analyses were performed. Results: 46 patients (mean age 51 years, 52% women, median dialysis vintage 46 months) had bone biopsies, DXA, and QCT scans. 37 patients (80%) had low bone volume by histology. DXA and QCT BMD values (g/cm^2^) were very highly correlated at the femoral neck (ρ = 0.97) and total hip (ρ = 0.97), and to a lesser degree at the spine (ρ = 0.65). DXA and QCT t-scores were also highly correlated, but QCT t-scores were systematically greater than DXA t-scores (1.1 S.D. on average at the femoral neck) leading to less recognition of osteopenia and osteoporosis by QCT. A t-score below –1 by DXA at the femoral neck (i.e., osteopenic or osteoporotic) showed 83% sensitivity and 78% specificity relative to low bone volume by histology. A QCT t-score below –1 did not reach acceptable diagnostic levels of sensitivity and specificity. Conclusions: DXA and QCT provide nearly identical areal BMD measures at the hip. However, QCT t-scores are consistently higher than DXA t-scores resulting in less diagnosis of osteoporosis or osteopenia. DXA results showed acceptable diagnostic sensitivity and specificity for low bone volume by histology and can be used for diagnosis of osteopenia and osteoporosis in patients with CKD-5D.

## Introduction 

Chronic kidney disease (CKD) is a pervasive health problem affecting more than 10% of the general population of the U.S., 31.7 million individuals [1, 2]. Chronic kidney disease-mineral and bone disorder (CKD-MBD) starts early during the loss of kidney function and is seen in virtually all CKD stage 5 patients [3]. Bone loss is an integral part of renal osteodystrophy, which encompasses the bone abnormalities of CKD-MBD [4, 5]. Bone mineral density (BMD) (gm/cm^2^) is generally considered the most important determinant of bone fragility to evaluate osteoporosis. It is of note that hip fractures occur in patients with CKD-MBD at a rate that is up to 10 times higher than in the general population [6, 7, 8, 9, 10, 11, 12, 13, 14]; with the associated high costs and morbidity. These fractures occur at an age 10 – 15 years younger than in non-CKD patients and have an annual subsequent mortality of 64% [8]. 

Dual-energy X-ray absorptiometry (DXA) is the most widely used tool for the assessment of bone mass and fracture risk in the general population. However, the Kidney Disease Outcomes Quality Initiative (KDOQI) recommends BMD measurements by DXA only in patients in which fractures have occurred [15], and the Kidney Disease Improving Global Outcomes (KDIGO) recommends explicitly against BMD measurements in CKD-3 to CKD-5D patients [16]. The data available to these organizations to make a recommendation about DXA utility were limited [17, 18]. Quantitative computed tomography (QCT) is an alternative to DXA that allows for the exclusion of the extraosseous calcification, but QCT has not been routinely used in CKD-5 patients for the measurement of BMD. In the general population DXA and QCT have both been shown to be useful in sensitivity and specificity for detecting osteoporosis [19, 20], and three-dimensional QCT is better in the spine than DXA and two-dimensional QCT at predicting osteoporosis or its sequelae [21, 22, 23]. 

Direct tissue analysis of bone volume can be made invasively by bone biopsies, but bone biopsies are rarely performed clinically due to the invasive nature of the procedure, and there are few laboratories able to process bone specimens without removal of bone mineral. 

This study was done to compare BMD measurements by DXA and QCT with validation using bone histology in CKD-5D patients. 

## Materials and methods 

### Patients and study protocol 

Patients were consented and enrolled into a prospective IRB approved study. The investigators adhered to the Declaration of Helsinki in the conduct of the study and registered the study with ClinicalTrials.gov (NCT00859612). Inclusion criteria were: age > 18, CKD-5D of at least 3 months duration, mental competence, willingness to participate in the study, and calcidiol levels within the normal range. Exclusion criteria were: pregnancy, systemic illnesses or organ diseases that may affect bone (except diabetes mellitus), clinical conditions that may limit study participation (e.g., respiratory distress and infections), chronic alcoholism and/or drug addiction, participation in a study of an investigational drug during the past 90 days, planning to move out of the area within 1 year, on active transplant list, and treatment within the last 6 months with drugs that may affect bone metabolism except for routine dialysis medications such as vitamin D, phosphate binders, and calcimimetics. 

### Assessment of bone volume and measurement of bone mineral density 

All patients had iliac bone biopsies. Bone samples were processed without removal of the mineral as described before [24, 25]. Briefly, bone samples were fixated in 100% ethanol, dehydrated, and embedded in methyl methacrylate. Sections of 4  µm were obtained and stained with modified Masson-Goldner trichrome. Sequential 7 µm sections were used for fluorescent microscopy. Bone samples were assessed qualitatively by two independent experienced bone pathologists (HHM and M-CM-F) who evaluated cancellous bone volume, cortical thickness, and cortical porosity in their assessment of low bone volume. The qualitative approach was chosen because that is the method used in daily clinical practice by pathologists. The inter-rater agreement rate for these two pathologists is 97.8%. Disagreements were discussed until consensus was reached. 

At the time of the bone biopsy, or shortly after, patients also underwent BMD scans of the lumbar spine and hip by QCT and DXA. Scans were performed using both methods by the same operator using the same machines for the duration of the study. iDXA (GE Medical Systems Lunar, Madison, WI, USA) was used for DXA; the coefficients of variation for DXA measurements were: spine 1.35% and hip 0.52%. QCT scans were performed on a SOMATOM Sensation 64 machine at 120 kVp with 2 mm slice thickness. Images were analyzed using QCT PRO software (Mindways Software Inc., Austin, TX, USA). The precision of QCT measurements was 3 mg/cm^3^. 

BMD absolute measurements were calculated as the average of the L1 through L4 values and the average of the bilateral femoral neck sites and total hip regions. A diagnosis of osteoporosis (t-score ≤ –2.5) or osteopenia (t-score > –2.5 but ≤ –1) was assigned to the patients using the lowest possible value from each site or region. QCT hip and spine measurements included both two-dimensional representations similar to DXA and three-dimensional scores of bone mass. 

### Statistical analysis 

Relationships between DXA and QCT areal measurements and t-scores were assessed using Pearson’s correlations (ρ) and linear regression. T-score distributions were compared graphically between sites and methods. Receiver operating characteristics curve analyses were performed to assess sensitivity, specificity, and area under the curve (AUC) by site and method relative to low bone volume by histology. SPSS^®^ version 22 (IBM^®^ Corp., Armonk, NY, USA) was used for all statistical calculations. 

## Results 

A total of 46 patients had bone biopsies and BMD scans between March 2009 and February 2014. There were 22 men and 24 women; 25 of them were black, 20 white and 1 of Asian race. The mean age was 50.7 years (standard daviation (SD) 14.0, range 21 – 82). Median dialysis vintage was 46 months (range 28 – 77 months). Clinical characteristics and treatment modalities are shown in Table 1. They are reflective of the general CKD-5D patient population. 

### Bone mineral density 

**Figure 1.**
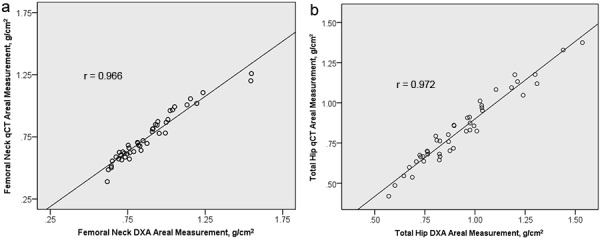
Dot graph of DXA and QCT areal measurements (g/cm^2^) of the femoral neck and total hip with regression lines.

DXA and QCT scans were performed on average within 1 month of the biopsy. Results showed a very strong linear correlation between DXA and QCT BMD measurements (g/cm^2^) at the femoral neck (ρ = 0.97, p < 0.001) (Figure 1a) and total hip (ρ = 0.97, p < 0.001) (Figure 1b) and less between the volumetric QCT measurements (mg/cm^3^) and DXA BMD measurements (g/cm^2^) at the spine (ρ = 0.65, p < 0.001). 

**Figure 2.**
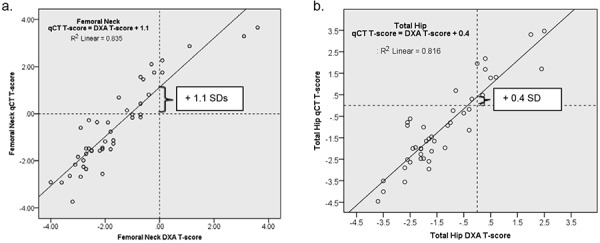
Dot graph of DXA and QCT t-scores of the femoral neck and total hip with regression lines.

**Figure 3:**
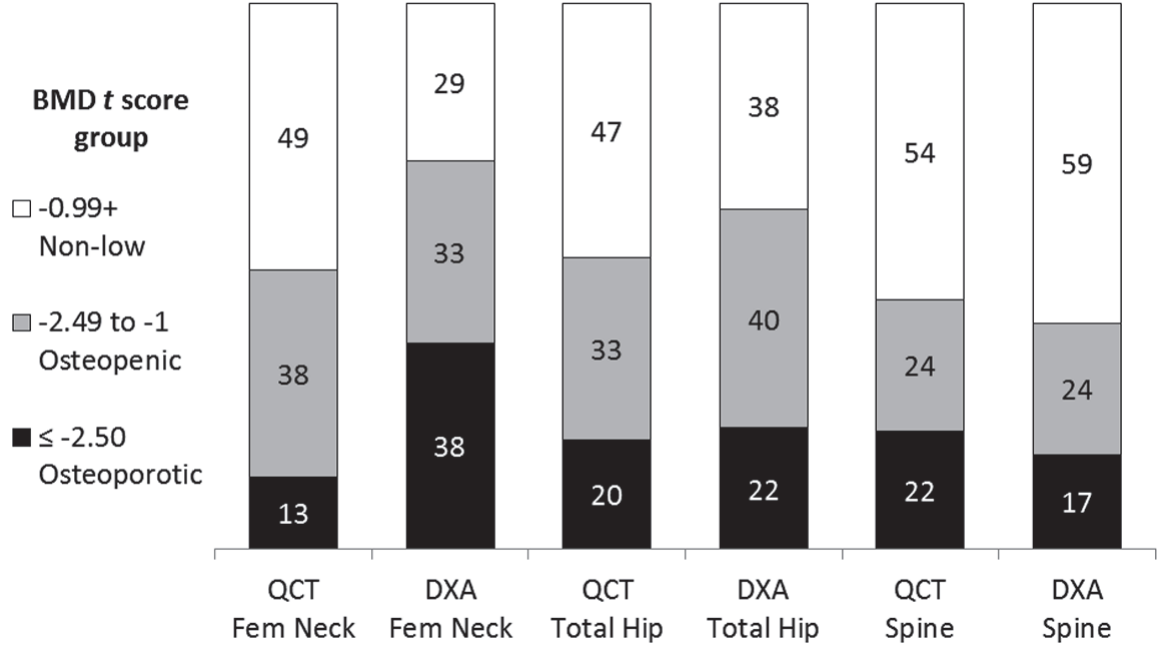
Percent of CKD-5D patients with osteopenia or osteoporosis by DXA or QCT at different sites.

DXA and QCT t-scores also correlated very highly at the femoral neck (ρ = 0.91, p < 0.001) (Figure 2a) and total hip (ρ = 0.90, p < 0.001) (Figure 2b) and less at the spine (ρ = 0.52, p < 0.001). However, regression analysis showed a significant shift upward in QCT t-scores relative to DXA t-scores of 1.1 SDs at the femoral neck (95% CI 0.8 – 1.4, p < 0.001) (Figure 2a); 0.4 SDs at the total hip (95% CI 0.1 – 0.7, p = 0.013) (Figure 2b) and 1.0 SDs at the spine (95% CI 0.1 – 0.7, p = 0.013). The higher QCT t-scores at the femoral neck and total hip resulted in QCT diagnosing fewer patients with osteopenia and osteoporosis than DXA (Figure 3). At the spine, QCT and DXA diagnosed similar levels of osteopenia and osteoporosis. 

### Diagnosis of low bone volume 

37 of the 46 patients (80%) were diagnosed with low bone volume by histology. Diagnosis of low bone volume was based on qualitative assessment of cancellous bone volume, cortical thickness, and cortical porosity. There were no differences in diagnosis of low bone volume by age (87% in patients > 50 years of age, 74% in those ≤ 50 years, p = 0.459); gender (73% in males, 88% in females, p = 0.276); race (76% in Black or African-Americans, 86% in Whites or Asians, p = 0.478); or dialysis vintage (77% in those > 46 months, 83% in those ≤ 46 months, p = 0.722). No osteomalacia was found. 

Sensitivity, specificity, and AUC calculations of BMD t-scores ≤ –1 for low bone volume by histology are shown in Table 2. A DXA t-score ≤ –1 at the femoral neck showed acceptable diagnostic precision for low bone volume by histology (sensitivity 83%, specificity 78%, AUC 0.81). A DXA t-score ≤ –1 at the femoral neck, total hip, or spine had a sensitivity of 89% and AUC of 0.83. A t-score ≤ –1 by QCT did not reach acceptable diagnostic precision for histologically determined low bone volume. 

## Discussion 

The presented data confirm that areal BMD measurements (g/cm^2^) using QCT are essentially identical to DXA at the hip [22]. However, when t-scores are used for assessment of results, there is an upward shift in the QCT normative curve compared to DXA. This shift resulted in QCT diagnosing less osteoporosis and osteopenia than DXA in this head-to-head comparison in CKD-5D patients. This discrepancy in detection of osteoporosis using t-scores was also noted in a prior study evaluating BMD [11] and is apparently due to differences in the normative databases used to derive the t-scores. We know that the DXA normative database was obtained from a wide range of individuals [26]. In contrast, there is limited information on the demographics of the QCT normative database. Khoo et al. [22] showed a similar agreement between QCT and DXA areal BMD measurements in 91 elderly women of the total hip and a similar shift of t-scores compared to what we observed. However, at the femoral neck, they observed a much smaller shift in t-scores than we did in our CKD-5D patients. This may be related to differences between patients with CKD-5D and the community volunteers studied by Khoo et al. [22]. 

Abnormal bone volume is an integral part of renal osteodystrophy which also encompasses mineralization and turnover abnormalities [4, 5]. For assessment of renal osteodystrophy, bone histology is considered the “gold standard” by KDOQI guidelines [15]. The mineralization and turnover abnormalities of renal osteodystrophy cannot be adequately assessed by DXA. This study evaluates the utility of DXA for the identification of renal osteodystrophy volume abnormalities. For this purpose we compared BMD measurements to histologically assessed low bone volume. Our data showed that a t-score ≤ –1 by DXA (that includes osteopenic and osteoporotic patients) at the femoral neck had acceptable diagnostic sensitivity and specificity for diagnosis of low bone volume by histology. This indicates that a t-score of ≤ –1 is an acceptable screening cutoff for low bone volume in patients with CKD-5D at which patients may undergo treatment. The usual t-score cutoff of –2.5 for diagnosis of osteoporosis misses too many patients with low bone volume, that is, it provides high specificity but low sensitivity. KDOQI guidelines do not recommend use of DXA for assessing bone mass and bone loss primarily because at the time the guidelines were established there was no data relating fracture risk to BMD in CKD. When our histologic data and the 71% prevalence of osteopenia or osteoporosis found in the current study are combined with a recent report demonstrating increased fracture risk in patients with lower BMD by DXA [27, 28, 29], there should be sufficient support to reconsider the current guidelines against using DXA or QCT for osteoporosis screening in CKD-5D patients. 

Given the extremely high correlation found in this study between DXA and QCT BMD areal absolute measurements at the hip, one might consider QCT to be equally useful for screening. However, the described t-score differences reduce QCT’s sensitivity relative to low bone volume, and when combined with the radiation exposure, do not justify its use for osteoporosis screening in CKD-5D patients. We also have previously shown that DXA detects more osteopenia [11]. 

Results were more complex at the lumbar spine where we did not observe significant t-score differences between DXA and QCT, and t-scores were generally higher for both methods than at the hip. This could be due to extraosseous calcifications detected by DXA counterbalancing higher t-scores by QCT, which only measures cancellous bone in the spine. Neither DXA nor QCT t-scores demonstrated diagnostic precision for low bone volume by histology. 

A limitation of this study is the relatively small sample size and its cross-sectional design. However, the invasiveness of bone biopsies limits its application, and this is a reasonable size for a direct comparison of established methods. Another theoretical limitation would be the use of qualitative assessment of cancellous and cortical bone mass by histology and not histomorphometric measurements. However, in routine practice, bone biopsies are assessed qualitatively and not by histomorphometry. This study was designed to reflect the clinical situation pertaining to workup in CKD-5D, and qualitative assessments have been shown to be in good agreement with histomorphometric results [24]. One could argue that iliac bone samples are not representative of systemic skeletal changes; however, this is repudiated by several comparisons of the iliac crest to other skeletal sites which demonstrated good correlations [29, 30, 31, 32, 33, 34]. In addition, Carvalho et al. [35] also showed that thoracic vertebral bone mass assessed by QCT correlates with iliac bone volume assessed by histomorphometry in hemodialysis patients. It is of note, they also found that up to 77% of hemodialysis patients had abnormalities by bone histomorphometry in the parameters we used for qualitative assessment of low bone volume. 

Even though there is no current FDA-approved treatment for osteoporosis in CKD-5D, greater awareness through non-invasive diagnosis should support the call for clinical trials evaluating treatment regimens; especially given the enormous morbidity, mortality, and costs caused by CKD-associated osteoporosis [28]. 

In conclusion, DXA and QCT provide virtually identical absolute BMD (g/cm^2^) measures at the hip. However, QCT t-scores are consistently higher than DXA t-scores which results in less diagnosis of osteoporosis or osteopenia. The shown limitations of QCT t-scores, combined with its radiation exposure, increased cost and reduced availability, render QCT not useful for screening purposes in these patients. DXA results showed acceptable diagnostic sensitivity and specificity for low bone volume by histology and can be used for diagnosis of osteopenia and osteoporosis in patients with CKD-5D. The affirmed usefulness of a commonly available screening tool should help overcome the current therapeutic neglect of CKD-associated osteoporosis, and should spur investigation into optimal treatment modalities. 

## Acknowledgments 

Research reported in this publication was supported by the National Institute of Diabetes and Digestive and Kidney Diseases of the National Institutes of Health under award number R01DK080770, and the Kentucky Nephrology Research Trust. The project was supported by the National Center for Research Resources and the National Center for Advancing Translational Sciences, National Institutes of Health, through Grant UL1TR000117. The content is solely the responsibility of the authors and does not necessarily represent the official views of the National Institutes of Health. We would like to thank Kimberly McLaughlin, Nedda Hughes, and Guodong Wang for their valuable technical assistance. 

## Conflict of interest 

The authors declare no conflicts of interest. 

**Table 1.** Patient clinical characteristics.

Number of patients	46
History of diabetes mellitus	17, 37%
History of coronary artery disease	12, 26%
Smoked within last two years	11, 24%
Medically treated hypertension	38, 83%
Treated with statins	19, 41%
Treated with cinacalcet	15, 33%
Treated with active vitamin D	22, 48%
Treated with calcium-containing phosphate binders	17, 37%

**Table 2.** Sensitivity and specificity of osteopenia or osteoporosis by DXA or QCT at the femoral neck, total hip, or L1 – L4 spine in detecting histologically determined low bone volume.

Diagnosis of osteopenia or osteoporosis by t-scores (BMD t-score ≤ –1)	Compared to low bone volume by biopsy		
Site and method	Sensitivity	Specificity	Area under ROC curve (95% CI)
Femoral neck DXA	83%	78%	0.81 (0.63 – 0.98)
Femoral neck QCT	58%	78%	0.68 (0.49 – 0.87)
Total hip DXA	72%	78%	0.75 (0.57 – 0.93)
Total hip QCT	64%	89%	0.76 (0.60 – 0.92)
L1-L4 spine DXA	47%	78%	0.65 (0.46 – 0.85)
L1-L4 spine QCT	53%	78%	0.63 (0.46 – 0.85)
DXA any site	89%	78%	0.83 (0.67 – 1.00)
QCT Any Site	72%	78%	0.75 (0.57 – 0.93)

DXA = dual-energy X-ray absorptiometry; QCT = quantitative computed tomography; BMD = bone mineral density; ROC = receiving operating characteristic; L1-L4 = lumbar vertebrae 1 through 4.
